# Editorial: Pathway of care and gaps in services for children and adults with autism spectrum disorder

**DOI:** 10.3389/fpsyt.2023.1193166

**Published:** 2023-04-13

**Authors:** Maria Luisa Scattoni, Andy Shih, Diana Schendel

**Affiliations:** ^1^Research Coordination and Support Service, Istituto Superiore di Sanità, Rome, Italy; ^2^Autism Speaks, Inc., New York, NY, United States; ^3^Department of Public Health, Aarhus University, Aarhus, Denmark; ^4^Lundbeck Foundation Initiative for Integrative Psychiatric Research, iPSYCH, Aarhus, Denmark; ^5^Department of Economics and Business, National Centre for Register-Based Research, Aarhus University, Aarhus, Denmark

**Keywords:** care pathways, services, care coordination, autism spectrum disorder, children, adults

This Research Topic entitled “*Pathway of care and gaps in services for children and adults with autism spectrum disorder*” aimed to provide evidence, data, or strategies for the definition and implementation of appropriate care pathways and services addressing the complex and heterogeneous nature of autism and providing high-quality, patient-centered medical care for autistic people. The pathway of care for autistic individuals is complex and should involve multiple systems, including healthcare, education, social services, and community support. The pathway begins with the diagnosis of autism, which can occur at any age but is typically diagnosed in early childhood. After the diagnosis, autistic individuals generally require ongoing support and care from multiple healthcare providers, including pediatricians, psychiatrists, psychologists, and other specialists. Their care should be personalized and, also, adapted to the cultural context. Access to early intervention programs is critical for improving outcomes for autistic children. As autistic individuals transition into adulthood, they may require additional support and services to help them achieve independence and integrate into the community. This Research Topic examines the journey of autistic individuals through the healthcare system and the available services and support. It also focuses on identifying gaps in the existing services and interventions for autistic individuals and how to address them to improve the quality of life for autistic people and their families. The specific barriers to accessing services in Africa, China, and Europe have been studied and analyzed by several authors on this Research Topic, which consists of 11 original articles, one systematic review, one review, one policy and practice review, and one mini-review, all published in 2022.

## 1. Low middle-income countries and minorities: diagnostic and intervention services

The definition, organization, and implementation of multidisciplinary care and effective coordination between different health/social care providers and services dedicated to children, adolescents, and autistic adults represent a real challenge, especially for low and middle-income countries. Pillay et al. examined providers' perspectives and offered solutions to meet the educational needs of autistic children by analyzing the data from semi-structured interviews in South Africa. The authors suggested learning from international best practices, developing long-term and integrated policies, including users and carers in the decisions, planning specific training programs for educators, supporting children, and enhancing the early diagnostic process (Pillay et al.). Liu et al. explored the intervention status and burden of autistic children in Guizhou, a region in the south of China, through a cross-sectional survey completed by 231 families with autistic children. The participants referred to a lack of intervention resources and the high psychological and economic burden of autism as the major challenge (Liu et al.). Borokova et al. performed a systematic investigation of the needs, access to services, and priorities of families of children with developmental disorders in Bulgaria using an online family survey; in the survey, 195 parents of autistic children and 73 parents of children with neurodevelopmental disorders (NDD) reported a lack of resources in services for diagnosis, treatment, and assistance. Autistic children needed different pathways compared to children with NDD and parents focused on the necessity of raising awareness of autism and protecting their children's rights (Borokova et al.). In their scoping review, Beauchamps et al. identified three levels of barriers to accessing services for minority-language speakers' families with children with NDD: (a) systems level, e.g., lack of high-quality treatment and training for healthcare professionals, difficulties accessing interpreters, and lack of available information in minority languages; (b) provider level, e.g., personal characteristics of healthcare practitioners or personal erroneous beliefs regarding language development; and (c) family experiences level, e.g., feelings of distrust in providers or feelings of stigma. Possible solutions proposed by the authors were: the development and uptake of policies and guidelines, practitioner's training, referral pathways for specialized services, access to appropriate tools and resources, and partnership with carers. Antony et al. showed, in their policy practice review, that the policy practices should support the conceptualization of autism in indigenous populations and their interaction with the Canadian health system and justice system. In addition, appropriate care strategies for this population should facilitate the coordination between health and social services and provide culturally appropriate multidisciplinary care (Antony et al.). Montenegro et al. conducted a study in Argentina stating that attention to the needs of autistic children has increased compared to a few years ago. For example, from 2015 to 2020, carers reported that the age of diagnosis of autistic children had decreased. However, some family members needed to leave their job to take care of their children. They also perceived that their rights should be protected and advocated for (Montenegro et al.). Zhang et al. described the use of a screening tool that combined the Modified Checklist for Autism in Toddlers, Revised with Follow-up (M-CHAT-R/F) and the Binomial Observation Test (BOT) in a resource-limited and highly populated Chinese context to improve early diagnosis in the routine 18- and 24-month of age visits. The study, conducted on a sample of 11,190 toddlers, showed that the diagnostic rate of autism through community screening was 0.32% (95% CI: 0.23–0.45%); the mean age at diagnosis for the children was 23.1 ± 4.55 months which was 20 months earlier than the autistic children not screened in the community screening program (Zhang et al.).

## 2. Autistic youths and adults: mapping of services and users' experiences

In 2017, the ASD in the European Union (ASDEU) conducted a survey for autistic adults, carers, and professionals exploring the services and practices needs of autistic adults (Micai et al.). The study reported the top choices by autistic adults, carers, and professionals for services best suiting their current needs as residential services, e.g., “help in own home” and “fulltime residential facility”; employment services: “job mentors” and “sheltered employment”; education services: “support in regular education setting”; financial services: financial support in lieu of employment or supplementing employment earnings for carers of highly independent adults and professionals; social services: “behavior training” and “life skills training.” The knowledge of good local service models that work well for autistic adults was generally low across all service areas (Micai et al.). Knowing where to find a specialized and dedicated service for autism is often one of the main barriers for adults and their carers. McCormick et al. described the “4-H,” a national inclusive program for life skills and transition for autistic youths. The authors conducted focus groups involving 20 educators that expressed the need to enhance training opportunities and resources.

## 3. The World Health Organization caregivers skills training: effectiveness, feasibility, and telemedicine

The World Health Organization Caregivers Skills Training (WHO CST) is an evidence-based treatment for families with autistic children. In this Research Topic, some studies focused on the effectiveness of the training and its application *via* telemedicine. Telemedicine improves access to services. Pacione et al. conducted a brief review of the application of the WHO CST for autistic children *via* telemedicine. Results showed a positive effect of the treatment in terms of feasibility, acceptability, and effectiveness. Montiel-Nava et al. tested the WHO CST in a rural community in Missouri, US, through an online group format for families with autistic children. The WHO CST was feasible and enhanced access to treatment and parental sense of competence. In addition, children showed fewer atypical behaviors and improvement in communication skills after the training. Finally, Lau et al. conducted a randomized comparison of the control waiting list and WHO CST (*via* eLearning asynchronous, videoconferencing with online coaching, and in-person hybrid modalities) in 34 Chinese dyads of parents and children with autism or other NDD. They showed that the WHO CST synchronous interaction, both online and in person, was effective in terms of children's clinical improvement of communicative skills (Lau et al.). An effectiveness and feasibility study of the WHO CST delivered and adapted in Taiwan revealed that this approach is positive in terms of the increase in knowledge and confidence of parents, and the reduction of the severity of autistic symptoms in children, with a maintenance effect, after 3 months (Seng et al.). Finally, Glumbic et al. showed the significant improvement in speech, language, and communication of the WHO CST program on children with developmental disabilities and evaluated the process of implementation, cultural appropriateness, and parental opinions on the program in Serbia.

## 4. Training of non-specialist personnel: promising resources and challenges

A scoping review by Naithani, Goldie, et al. on intervention approaches for families with autistic children in low and middle-income countries showed preliminary fidelity, acceptability, and effectiveness of delivering intervention elements and techniques within parent-mediated programs by non-specialist personnel (i.e., bachelor-level graduates with no prior exposure to child development or autism) delivered at home and in clinical settings, with a supervision team (Naithani, Goldie, et al.). However, in another study by the same authors, the training of non-specialist practitioners reported mixed results (Naithani, Sangwan, et al.). A task-sharing approach to support the delivery of parent-mediated treatment for autism in South Asia (COMPASS) tested in a group of non-specialist Indian workers by video training did not appear to be an effective method to ensure the motivation of participants (Naithani, Sangwan, et al.).

## 5. Conclusions

In summary, despite the availability of many services and interventions for autistic individuals, there are still significant gaps in care. These gaps include disparities in access to diagnosis and care, inadequate training for healthcare providers, and limited funding for research and services. One significant gap in care for autistic individuals is the lack of access to early diagnosis and intervention services, particularly in underserved communities. Many families face long waitlists and limited availability of specialized services, leading to delays in accessing critical care. Another significant gap in care for autistic individuals is the lack of training for healthcare providers in autism diagnosis and management. Many healthcare providers report feeling unprepared to provide care for autistic individuals and lack knowledge of evidence-based interventions. Identifying and addressing gaps in care is essential for improving outcomes for autistic individuals and reducing disparities in access to care. Further research is needed to understand the best practices for delivering care and improving outcomes for autistic individuals.

## Author contributions

MS wrote the first draft of the manuscript. All authors contributed to the conception of the Editorial. All authors contributed to the manuscript revision, read, and approved the submitted version.

